# Multidisciplinary approach of teaching radiology to medical students in Egypt: Is this an effective method?

**DOI:** 10.1186/s43055-021-00672-1

**Published:** 2021-12-07

**Authors:** Yasmeen Nabhani, Victoria K. Xie, Mohamed Badawy, Rehan Karim, Umayma Abdullatif, Ahmed S. Negm, Hrishabh Bhosale, Scott Rohren, Ahmed Elhatw, Sammar Ghannam, Abdelrahman Abusaif, Mazzin Elsamaloty, Nada Shalaby, Ferial Choucair, Islam Khalifa, Mariam Ahmed Saad, Parth Patel, Zaid Almubaid, Mostafa Ahmed Shehata, Yara ElHefnawi, Serageldin Kamal, Mahmoud F. Hammad, Khaled M. Elsayes

**Affiliations:** 1 Sinai Hospital of Baltimore, Baltimore, MD USA; 2 Baylor College of Medicine, Houston, TX USA; 3 The University of Texas MD Anderson Cancer Center, Houston, TX USA; 4 HCA Houston Health Care West, Houston, TX USA; 5 University of Houston, Houston, TX USA; 6 Mayo Clinic, Rochester, MN USA; 7 University of Texas at Austin, Austin, TX USA; 8 National Cancer Institute, Cairo University, Cairo, Egypt; 9 University of Texas San Antonio, San Antonio, TX USA; 10 College of Medicine and Life Sciences, University of Toledo, Toledo, OH USA; 11 Faculty of Medicine, Alexandria University, Alexandria, Egypt; 12 McGovern Medical School at UTHealth, Houston, TX USA; 13 University of Texas, Austin, TX USA; 14 Kasr Al-Ainy Medical School, Cairo University, Cairo, Egypt; 15 National Liver Institute, Menoufia University, Menoufia, Egypt

**Keywords:** Multidisciplinary, Radiology education, International, Undergraduate

## Abstract

**Background:**

In multidisciplinary education, different perspectives from more than one discipline are used to illustrate a certain topic. The aim of this study was to evaluate the effectiveness of an online, multidisciplinary radiology curriculum to teach radiology to medical students in Egypt. A multidisciplinary team of radiologists, surgeons, and internists taught a series of 5 case-based radiology sessions on a web conference platform. Topics included common clinical case scenarios for various body systems. Undergraduate medical students across Egypt were enrolled in the course. A pre-test–post-test design was used to evaluate the efficacy of each session. Upon course completion, students filled out a subjective survey to assess the radiology education series.

**Results:**

On average, 1000 students attended each session. For each session, an average of 734 students completed both the pre-test and post-test. There was a statistically significant increase in post-test scores compared to pre-test scores across all 5 sessions (*p* < 0.001) with an overall average score improvement of 63%. A subjective survey at the end of the course was completed by 1027 students. Over 96% of students found the lecture series to be a worthwhile experience that increased their imaging knowledge and interest in radiology, and that the use of a multidisciplinary approach added educational value. About 66% of students also reported that the session topics were “excellent and clinically important.” There was a marked increase in reported confidence levels in radiology competencies before and after attendance of the sessions.

**Conclusions:**

An online radiology curriculum with a multidisciplinary approach can be implemented successfully to reach a large group of medical students and meet their educational objectives.

## Introduction

Since March 2020, the COVID-19 pandemic has heavily impacted medical education around the world. Within Egypt, in-person undergraduate medical courses and clinical clerkships were suspended out of concern for the safety of students and teachers. As a result, Egyptian medical students have largely exchanged physical classrooms for online learning webinars [1]. Multiple studies, including radiology education-specific ones with small sample sizes, have implemented virtual medical courses (interactive web conferences) to assess student performance and satisfaction. These teaching modalities including the flipped classroom model, didactic lectures, case-based discussions/read-outs, online modules, interdisciplinary conferences, and small group activities have received mostly positive reviews [2–4].

Radiology, in particular, is well-positioned to be taught electronically due to its heavy reliance on technology and the existence of multiple online educational radiology module platforms [5]. Unfortunately, radiology has been historically underrepresented in the medical curriculum [6]. Despite medical imaging becoming an increasingly routine part of patient care, medical student radiology education has not achieved similar growth [6]. Radiology education in many medical schools is often not part of the required curriculum and is only offered in the forms of electives often taught by non-radiologists, such as anatomists [6, 7]. Effective radiology education not only exposes medical students to the field of radiology but also can inspire students to pursue careers in radiology and build a strong foundation of basic radiology knowledge [3].

In addition, many specialties (internal medicine, obstetrics-gynecology, pediatrics, general surgery) have reported that incoming interns have insufficient imaging interpretation skills (“normal vs abnormal”) and an inability to order imaging according to appropriate imaging guidelines. This is likely attributable to lack of radiology education in medical schools [8]. One possible solution to this problem is using a multidisciplinary approach to integrate direct radiology instruction by radiologists, supplemented by perspectives from other specialties. Integrating radiology education with topics across different specialties can help to increase understanding and appreciation of the role of radiology in multifaceted patient care [9]. Implementing a formal, multidisciplinary radiology education across medical curricula may also improve the retention of foundational radiology knowledge for both future radiologists and non-radiology physicians. In the following report, we strove to implement and assess a similar multidisciplinary online radiology educational series within a cohort of Egyptian medical students, a similar methodology to a previous, parallel United States (U.S.) study. We hypothesize that this virtual multidisciplinary radiology educational series can be used to meet the educational needs of a large group of medical students internationally.

## Methods

Participants of this study provided informed consent to authorize the use of the test results and the analysis of the survey answers for research purposes.

### Development and implementation

An online, multidisciplinary diagnostic radiology lecture series was designed to run for 5 sessions led by a radiologist, an internist, and a surgeon; they addressed the following topics: child abuse, bone fractures, breast, pneumonia, and flank pain (Table 2). The sessions were attended by 1363 medical students from Egyptian medical schools. Student ambassadors communicated the schedule from the course director to the remaining students and set up 5 live online sessions. The sessions were hosted on the video conferencing platform, Zoom (Zoom Video Communications Inc., 2016), which allows screen sharing in addition to audience interaction in the questions and answers session. The live sessions began with a 10-min multiple choice pre-test knowledge assessment. Then, the panelists would discuss a teaching topic, beginning first with the internist or surgeon who presented a case and then a radiologist would go over relevant imaging. The radiologist would describe the imaging studies that were ordered, the interpretation of the images, and how these images guided medical or surgical management. Imaging scans were presented both as static images and as videos to explain relevant pathology and normal anatomy. Once the lectures were completed, the students completed a post-test and then had a 15-min question and answer session with the panelists. A learning objective hand out was sent out to medical students few days after each live session to enhance the learning experience for students and provide them with educational material that they can reference.

### Evaluation

We implemented a correlational pre-test–post-test design to assess the change in knowledge gained by students through the virtual lectures. An assigned medical student helped by the panelists at each session to set up 7–13 questions multiple choice questions that covered the learning objectives of that session. The same set of questions was administered before and after the session using Qualtrics survey software (Qualtrics, Provo, UT) to automatically score each test, awarding one mark per correct answer. Identifying information was removed from each test using a deidentifier code assigned to each student. At the course’s conclusion, students completed a 17-question course evaluation survey. The questions evaluated students’ overall experience using open-ended feedback questions, an assessment of the confidence regarding their understanding of imaging studies both pre- and post-course, and rating each of the 10 sessions individually using the following point scale: poor = 1, fair = 2, good = 3, and excellent = 4.

### Recruitment and student leadership

The course director recruited ambassadors from medical students all over Egypt based on a survey they had filled out before the course start date. Ambassadors help to recruit potential participants and to facilitate the communication during the course and after the course. The ambassadors distributed the course details and registration Google Forms (Google, Mountain View, California) for the course to their schools’ group chats and Facebook groups to let interested medical students know about the course. The ambassadors played a key role organizing the course. In addition to recruitment, they actively contributed to the design of pre- and post-tests based on reading material provided by the instructors, preparation of the sessions’ handouts, and communicating attendance information with the attendees. The ambassadors had weekly meetings during the course at which they provided valuable input, directions for the course, and added to design of the final evaluation survey.

### Statistical analysis

We utilized a paired t-test within Excel (Microsoft, Redmond, Washington) to compare pre-test and post-test scores from students in each session separately. Unless a medical student completed both pre- and post-tests, the score of the exam was not included in the analysis. A *p*-value of less than 0.05 was used to indicate a statistically significant difference between pre- and post-test scores.

Following completion of all 5 sessions, participating students were asked to complete a subjective course evaluation survey assessing for changes in their confidence levels of their basic radiology skills, on a four-point Likert-type scale ranging from “not confident at all” to “very confident.” Using MATLAB (The MathWorks, Natick, Massachusetts), the Wilcoxon signed-rank test with a one-tailed hypothesis was performed to evaluate for statistically significant differences (*p*-value < 0.05) between confidence levels before and after the course.

## Results

A total of 1363 medical students enrolled in the radiology course after filling out an interest form and completing a perquisite online course about the fundamentals of imaging. On average, 1000 students attended each session. For each session, an average of 734 students completed both the pre-test and post-test.

In total, 1027 students completed the end-course survey. Of which, 66.5% were first through sixth-year medical students and 33.5% were interns or non-radiology house staff. The gender distribution was 56.3% female, and 0.2% preferred not to answer. The main language used to teach medical students at their school was overwhelmingly English (91.1%). Table 1 displays student demographics.

**Table 1:** Demographics

	%	*N*
Medical school class		
First-year	1.07	11
Second-year	4.48	46
Third-year	7.89	81
Fourth-year	12.27	126
Fifth-year	18.99	195
Sixth-year	21.81	224
Other	33.50	344
Gender		
Male	43.33	445
Female	56.48	580
Other	0.00	0
Preferred not to answer	0.19	2
Language		
English	91.14	936
Arabic	7.79	80
Other	1.07	11

**Fig. 1:**
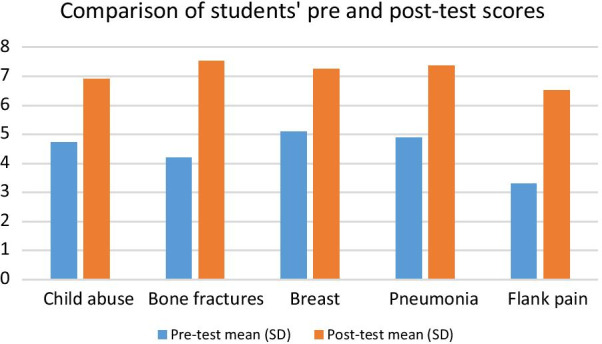
Comparison of students' pre- and post-test scores

The number of students who completed both pre- and post-tests was the highest at the third lecture (794 students). Improvements in students’ post-test scores were noted compared to their pre-test score (Fig. 1). The highest improvement in students’ acquisition of knowledge was in the last lecture (97% change). Across all 5 sessions, there was a statistically significant increase in post-test scores compared to pre-test scores (*p* < 0.001) with an overall average score improvement of 63% (Table 2).

**Table 2:** Pre- and post-test scores by session

Session	Completed tests	Pre-test mean (SD)	Post-test mean (SD)	% Change	*p* value
Child abuse	735	4.73 (1.43)	6.91 (1.78)	46	< 0.001
Bone fractures	758	4.20 (1.53)	7.53 (2.03)	79	< 0.001
Breast	794	5.08 (1.87)	7.25 (2.10)	43	< 0.001
Pneumonia	682	4.89 (1.64)	7.36 (2.01)	50	< 0.001
Flank pain	703	3.31 (1.82)	6.52 (2.72)	97	< 0.001

Most students (over 96%) reported that the lecture series was a worthwhile experience that strongly or somewhat increased their knowledge of imaging as a diagnostic tool, as well as their overall interest in radiology. About 91% strongly or somewhat agreed that the topics presented were relevant to their medical education. Greater than 93% of participants stated the course met their expectations and found the course material to be just right in difficulty level and amount of effort to complete. Ninety-four percent of attendees strongly or somewhat agreed that the use of a multidisciplinary approach with the additional presence of a non-radiologist, such as a surgeon or internist, added educational value.

The weighted average rating of all 5 sessions was 3 points or “good.” On average 50.8% of participants rated each session as “excellent,” 39.1% as “good,” and 9.3% as “fair.”

**Fig. 2:**
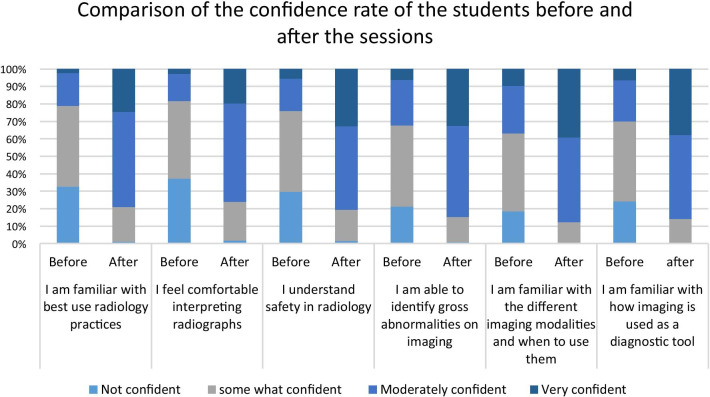
Comparison of the confidence rate of the students before and after the sessions

Subjective assessment of the students’ confidence levels in different radiology competencies was measured through the end-course survey. While most of the students before the course chose “not confident” or “somewhat confident” in the assessed radiology competencies, most students selected “moderately confident” or to a lesser extent, “very confident” after completing the course. Figure 2 summarizes the participants’ confidence level ratings before and after the course. Across all five sessions, a statistically significant increase was noted in the students’ confidence level after completing the course compared with their confidence levels before (all *p* < 0.001). (Table 3).

**Table 3:** Statistical analysis of the confidence level of the students before and after the sessions

Session	Weighted average before the session	Weighted average after the session	*p* value
I am familiar with best use radiology practices	1.91	3.02	< 0.001
I feel comfortable interpreting radiographs	1.84	2.94	< 0.001
I understand safety in radiology	2	3.12	< 0.001
I am able to identify gross abnormalities on imaging	2.17	3.16	< 0.001
I am familiar with how imaging is used as a diagnostic tool	2.28	3.26	< 0.001
I am familiar with the different imaging modalities and when to use them	2.12	3.23	< 0.001

## Discussion

The COVID-19 pandemic forced educators to find a balance between providing enriched learning experiences for their students while also adhering to public safety measures. Virtual learning models were utilized as medical schools halted in-person learning for several months during the height of the pandemic in the Spring of 2020 [10].

The field of radiology lends itself well to virtual education [11]. Readouts performed over virtual platforms, didactic lectures given in live and prerecorded formats, and interactive methods such as audience polling and quizzes were successful alternatives when in-person meetings were not possible [12].

In implementing a virtual radiology rotation for medical students in Egypt, we assessed pre-test and post-test data for five different radiology diagnoses. In comparing pre-test and post-test results, the cohort of students performed better on the post-test after completing the virtual rotation, with statistically significant results. In the same fashion, several studies showed the effectiveness of virtual teaching of radiology [2, 13]. In a study from Harvard Medical School, 111 medical students enrolled in a virtual radiology curriculum and were given a final exam at the conclusion of the rotation. The final exam results were compared to those of the final exam taken when the rotation was in-person in prior years, with the final exam scores being similar to those of the in-person rotation. This demonstrates the quality of online education delivery with minimal to no deficiency when compared to in-person learning [13]. In the Egyptian cohort that we assessed, employing a similar comparison model with the performance of students enrolled in an in-person radiology curriculum at Egyptian medical schools pre-COVID-19 pandemic could be useful to assess efficacy for future implementation of online learning.

An overwhelming majority of the participants felt that the virtual program increased their interest in radiology as well as their knowledge of using radiology as a diagnostic tool in medicine. With a larger percentage of participants in the Egyptian cohort being undergraduate medical students at Egyptian medical schools, tailoring the difficulty level of the program to match that of a student was key. This was successful as demonstrated by post-course survey results that the material and topics were “just right” and clinically important.

A number of subjective points were assessed pre- and post-course, including familiarity with radiology best-use practices, identifying gross abnormalities on imaging, and the utility of different imaging modalities in different clinical scenarios. The finding of our results is consistent with other studies and supports the value supplemental radiographic education may provide in improving the competency of clinicians and medical students in their specialty [14–17].

In addition, using an online educational model also helps reach a greater number of students and gives them access to the same kinds of resources, which offers a robust learning experience and more equal field of opportunities that these students may otherwise not have been able to access [18, 19]. This is shown in other virtual education models that have been implemented in interdisciplinary fields in Egyptian medical schools. In medical schools in Assiut, Egypt, flipped classrooms, virtual small group sessions, and the use of simulation videos to teach procedural clinical skills were implemented. Moreover, students were incorporated in telehealth clinic visits with patients, which is helpful given the dynamic practice of medicine [14].

A large majority of the participants agreed to varying extents that the presence of an internist or surgeon at the educational sessions added value to the program. These results were similar to another study that reported the effectiveness of multidisciplinary approach in incorporating radiology in anatomy education [20, 21]. The approach to patient care and treatment continues to grow in a collaborative model. Introducing radiology at an early stage of medical training may reinforce a superior approach to patient care as future healthcare professionals [22]. In addition, learning the radiographic significance of certain pathological diseases can serve as a proactive way for students to correlate better with clinical presentation.

Overall, employing a multidisciplinary approach to radiology education at the medical student level can successfully be done virtually, both in response to the COVID-19 pandemic as well as to the changing fabric of medicine, with telehealth becoming more popular [18, 23]. Online medical education can reach a wider range of students and their learning can be adequately assessed with a combination of live discussion sessions and testing methods [19, 24].

### Limitations

As our study depended on students answering both tests, students who answered only one were not included, thus preventing us from attaining a 100% response rate. However, online surveys are expected to not reach a 100% response rate, and we received an appreciable percentage of an ~ 80% response rate for both pre-test and post-test.

## Conclusions

An online multidisciplinary approach to teaching radiology to medical students can effectively and efficiently meet medical education needs. The accessibility, ease of information delivery, and overall well-received curriculum demonstrates that even beyond the circumstances presented by the COVID-19 pandemic, online learning models may be incorporated more permanently in medical education and reach even more students at other institutions.

## Take-home points

- It is practical to implement radiology education via online learning platforms.
- Online and virtual video conferencing platforms that are available and established make it possible to broaden the use of educational resources, including access to professors from different fields and countries, FOAMed (Free Open Access Medical Education) in order to create a rich learning experience despite being completely virtual.
- A multidisciplinary approach in teaching radiology showed significant increase in knowledge gained by students of the targeted teaching topics regarding basic radiology interpretation skills and understanding imaging.
- Multidisciplinary education that incorporates radiology plays an important role in diagnostic medicine and medical management.

## Data Availability

Not applicable.

## Abbreviations

U.S.: United States
FOAMed: Free open access medical education
